# The multiple roles of microRNA-155 in oncogenesis

**DOI:** 10.1186/2043-9113-3-17

**Published:** 2013-09-28

**Authors:** Gadareth Higgs, Frank Slack

**Affiliations:** 1 Yale Center for Medical Informatics, Yale University, Suite 505, 300 George Street, New Haven, USA; 2 Department of Molecular, Cellular and Developmental Biology, Yale University, KBT 936, PO Box 208103, New Haven, CT 06520, USA

**Keywords:** miR-155, Oncogenesis, OncomiRs, Anti-miR therapy, Nanoparticle delivery

## Abstract

The microRNA miR-155 is prominent in cancer biology. Among microRNAs that have been linked to cancer, it is the most commonly overexpressed in malignancies (PNAS 109:20047-20052, 2012). Since its discovery, miR-155 has been implicated in promoting cancers of the breast, lung, liver, and lymphatic system. As such, targeted therapies may prove beneficial to cancer treatment. This review discusses the important role of miR-155 in oncogenesis. It synthesizes information from ten recent papers on miR-155, and includes an analysis and discussion of its association with cancer, interactions with other miRNAs, mechanisms of action, and the most promising available treatment options.

Current debates in the field include the importance of miRNAs in general and their utility as targets in preventing tumorigenesis (Blood 119:513-520, 2012). Most of the papers being reviewed here confirm the role of miR-155 in oncogenesis (EMBO Mol Med 1:288-295, 2009). While there is some controversy surrounding recent research that claims that miR-155 may display anti-oncogenic or pro-immunological benefits (Cell Rep 2:1697–1709, 2012), most research seems to point to the importance of anti-miRs, with anti-miR-155 in particular, for cancer therapy.

## Introduction

MicroRNAs (miRNAs) are short (about 21 nucleotides long) strands of non-coding RNA that regulate the expression of multiple genes [1]. They are important post-transcriptional regulators of gene expression in plants, metazoans, and mammals, and are predicted to control the activity of 30% of all protein-coding genes [2]. MiRNAs control cell functions by silencing genes or gene clusters and may inhibit RNA translation by mRNA uncapping and deadenylation, which lead to increased mRNA turnover and decreased target gene expression [3].

Because of their wide variety of targets, miRNAs have been found to affect numerous developmental processes within cells including hematopoietic lineage differentiation, immunity, inflammation, and tumorigenesis [4,5]. Those miRNAs that lead to tumorigenesis and cancer are classed as oncomiRs. These oncomiRs are not only therapeutic targets, but also important biomarkers for cancer detection and management [6].

Among the known oncomiRs, miR-155 stands out as an important entity [7]. It is one of the most commonly up-regulated miRNAs in solid and hematological malignancies [8], and has been linked to the development of leukemia, breast, lung, and stomach tumors [9]. As such, it is an important target for diagnosis, prognosis [10], and therapy. While experimental evidence has shown that miR-155 is over-expressed in malignant tumors, the specific mechanism of action of miR-155 was unknown until recently.

MiR-155 is produced from the processing of the B-Cell Integration Cluster (BIC), which is a non-coding transcript expressed in activated B cells, T cells, monocytes and macrophages [11,12]. The BIC gene is activated by promoter insertion at a retroviral integration site on chromosome 21q21 in B cell lymphomas induced by avian leukosis virus. Knockouts of miR-155 show defects in hematopoietic development [13].

## Review

### What are the physiologic and oncogenic functions of miR-155?

Cancer is marked by six hallmarks:

1. Maintaining proliferative signaling

2. Evading growth inhibitors

3. Resisting cell death

4. Enabling replicative immortality

5. Inducing angiogenesis

6. Activating invasion and metastasis

As a prominent oncomir, miR-155 plays a role in many of the above oncogenic processes. It down-regulates BCL6 (B-cell lymphoma 6) protein [8], which is an evolutionarily conserved zinc finger transcription factor that contains an N-terminal POZ/BTB domain. As BCL6 has been shown to modulate the STAT-dependent Interleukin 4 (IL-4) responses of B cells, miR-155 acts to increase B cell functioning. Also, since BCL6 interacts with several co-repressor complexes to inhibit transcription, and its gene is frequently trans-located and hyper-mutated in diffuse large B cell lymphoma (DLBCL), miR-155 acts to enhance transcription and contribute to the pathogenesis of DLBCL. Reduction of BCL6 then leads to up-regulation of known BCL6 targets such as inhibitor of differentiation (Id2), Interleukin-6 (IL6), cMyc, Cyclin D1, and Mip1α/Ccl3, all of which promote cell survival and proliferation [8].

MiR-155 also upregulates Mxd1/Mad1, a network of basic helix-loop-helix leucine zipper transcription factors which mediate cellular proliferation, differentiation, and apoptosis, through regulating BCL6. In this way, MiR-155 leads to the resistance of cell death and enables replicative immortality.

HDAC4 (histone deacetylase 4), a co-repressor partner of BCL6, is a direct target of miR-155. As histones play a critical role in transcriptional regulation, cell cycle progression, and developmental events, HDAC4 alters chromosome structure and affects transcription factor access to DNA. It does not bind DNA directly, but attaches through the transcription factors MEF2C and MEF2D. Analysis of DLBCL patient data showed that miR-155 expression is inversely correlated with Bcl6 and HDAC4 [8]. Additionally, miR-155 was upregulated in Hodgkin, primary mediastinal B-cell lymphoma, and DLBCL [8].

Mir-155 was also shown to down-regulate HGAL by binding to its 3′ UTR, leading to decreased RhoA activation and increased spontaneous and chemoattractant-induced lymphoma cell motility [14]. MiR-155 directly increased TNF-α levels by augmenting transcript stability through binding of its 3′ UTR. MiR-155 also targets gene transcripts coding for proteins that are known to be TNF-α translation repressors [4], and evades growth inhibitors by targeting SH2-containing inositol phosphatase (SHIP1), a negative regulator of myeloid cell proliferation and survival [15].

Hexokinase 2 (hk2) is up-regulated by miR-155 through two distinct mechanisms. Firstly, miR-155 promotes hk2 transcription by activation of signal transducer and activator of transcription 3 (STAT3), a transcriptional activator for hk2. Secondly, by targeting C/EBPβ (a transcriptional activator for mir-143), miR-155 represses mir-143, a negative regulator of hk2, thus resulting in up-regulation of hk2 expression at the post-transcriptional level [16].

The suppression of miR-155 inhibited cell proliferation and migratory activity and induced apoptosis in renal cancer cells. The suppressor gene suppressor of cytokine signaling (SOCS-1) and BACH1 were predicted as potential target genes by bioinformatics analysis. The suppression of miR-155 inhibited BACH1 protein expression. Therefore, it was determined that miR-155 may function as an oncogene by targeting BACH1 [17].

In the Leukemia paper by Forrest et al., Mir155 was found to induce G2 arrest [18]. Ectopic miR155 expression in mice B cells induced pre-B cell proliferation followed by high-grade lymphoma/leukemia [8].

MiR-155 (along with miR-125b) was also a contributor to BCL2 repression and proliferation in response to CD40 ligand (CD154) in human leukemic B-cells [19]. MiR-155 also targeted casein kinase 1α (CK1α), which enhanced β-catenin signaling and cyclin D1 expression, thereby promoting tumor cell growth [3]. In the Willimott and Wagner paper, it was shown that MiR-155 repressed BCL2 mRNA. This required CD154, which resists cell death.

**Figure 1:**
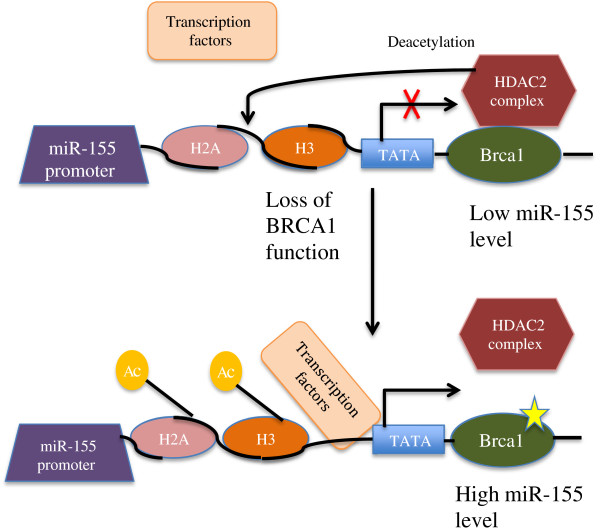
**Normal BRCA1 represses miR-155 expression via its association with HDAC2 [**[20]**].**

It has also been shown that miR-155 is epigenetically repressed by BRCA1 (breast cancer type 1 susceptibility protein). This occurs via an association with HDAC2, which deacetylates H2A and H3 on the miR-155 promoter [20], and is shown in Figure 1 below.

### What is miR-155′s association with cancer?

In a paper by Pedersen et al., miR-155 was shown to be an oncomiR whose expression in B cells alone triggers malignant transformations [21]. The authors studied the effect of miR-155 in diffuse large B-cell lymphoma (DLBCL) and identified the SH2 domain containing inositol-5-phosphatase (SHIP1) as a target of miR-155. SHIP1 is a hematopoietic cell protein whose movement, from cytosol to plasma membrane, is mediated by tyrosine phosphorylation.

It was demonstrated that DLBCL cells showed elevated levels of miR-155 and diminished levels of SHIP1 [21]. This was believed to have been the result of autocrine stimulation by cytokine TNF-α. The authors showed that the administration of anti-TNF-α regimen with eternacept or infliximab decreased miR-155 levels and restored SHIP1 expression. Additionally, cellular proliferation was decreased along with tumor size, showing that cytokine-regulated miRNAs, such as miR-155, are a critical link between inflammation and cancer. This paper was important because anti-TNF-α therapy for DLBCL was both novel and easily accessible. As DLBCL accounts for about 40% of adult non-Hodgkin lymphomas, this research has important applications for cancer treatment.

While there is overwhelming evidence that miR-155 expression is linked to lymphatic cancers [8,14,15,21-23], in their 2012 paper, Pingyu Zhang and fellow researchers at The University of Texas MD Anderson Cancer Center showed that miR-155 also plays a role in liposarcoma [3]. In this study, the authors studied the expression profile signature of 35 miRNAs and found that miR-155 was the most overexpressed. They found that this miRNA was integral in the growth of dedifferentiated liposarcoma cell lines and that miR-155 knockdowns induced G_1_S cell-cycle arrest, inhibited tumor cell growth, and had decreased colonies. The researchers also determined that miR-155 targeted casein kinase 1-α (CK1-α), enhancing Beta-catenin signaling and cyclin D1 expression, thereby promoting tumor cell growth.

This study was significant because it showed that miR-155 has a role in mesenchymal solid malignancy. Previously, miR-155 had been implicated in the promotion of hematologic and epithelial cancers.

Much of the current research in the field has implicated miR-155 in promoting oncogenesis. However, in their recent Blood paper, Zonari et al. studied the anti-oncogenic effects of miR-155 [24]. They discovered that miR-155 knockdown in myeloid cells facilitated breast cancer development in mice. It was shown that miR-155 was required by tumor-associated macrophages (TAMs) for anti-tumoral activity. They determined that the miR-155 knockdown promoted tumor growth by impairing classical activation of tumor-associated macrophages (TAMs). This, in turn, led to a reduction in CD11c^+^ TAMs, reduced expression of activation markers, and a skewing of tumor immune cells to an M2/Th2 response. Their research was done in MMTV-PyMT mice, which is a mouse model that closely mimics tumor-host interactions seen in humans. While this research could be applicable to *Homo sapiens*, the authors themselves noted that results generated in mice might not apply to humans [24]. Therefore, more research must be done in this arena before one can translate these results into clinical trials.

The preceding papers show that miR-155 plays an integral role in lymphatic cancer, liposarcoma and breast cancer by affecting different targets. Table 1 below summarizes many of the known important targets of miR-155. In propagating lymphatic cancer, miR-155 is upregulated by the cytokine TNF-α, and targets SHIP1, an important protein for myeloid cell proliferation and survival. In liposarcoma, miR-155 targets CK1-α to enhance Beta-catenin signaling and cyclin D1 expression and promote tumor cell growth. In breast cancer, miR-155 associates with TAMs, which prevent tumor growth, and therefore seems to prevent breast cancer in mice. The table also shows that miR-155 targets molecules that play a role in pancreatic cancer, colon cancer, leukemia, and renal cancer. In each study, miR-155 interacts with proteins and other molecules in order to affect the progression to cancer. However, miR-155 also associates with other miRNAs, and in the next section, these associations will be explored.

**Table 1:** MiR-155 target genes & associations

**MiR-155 target**	**Association**
**SHIP & C/EBP-Beta**	B-cell malignancies [3]
**TP53INP1**	Pancreatic Cancer [3]
**FOXO3**	Secondary Acute Leukemia (MLL) [3]
**RHOA**	Breast Cancer [3]
**MSH2**	Colon Cancer [3]
**MSH6**	Colon Cancer [3]
**MLH1**	Colon Cancer [3]
**SOCS1**	Hodgkin and B Cell Lymphoma [3]
**Meis1**	Not-oncogenic [3]
**c-MAF**	Not-oncogenic [3]
**AID**	Not-oncogenic [3]
**IL-1**	Not-oncogenic [3]
**IKK-epsilon**	Not-oncogenic [3]
**ETS-1**	Not-oncogenic [3]
**BACH1**	Renal Cancer [17]

### Interaction with other miRNAs

MiR-155 has been shown to interact with other miRNAs in the regulation of cancer [16]. In their 2012 paper, Jiang and fellow researchers discovered a novel miR-155/miR-143 cascade that controlled glycolysis in breast cancer cells. They found that miR-155 was induced by inflammation, and it mediated cytokines, which promoted glycolysis. MiR-155 was found to up-regulate HK2 by activating Signal Transducer and Activator of Transcription 3 (STAT3), and targeting C/EBP-β (CCAAT/enhancer-binding protein beta: a transcription activator for miR-143). Therefore, upregulation of miR-155 led to miR-143 repression and HK2 up-regulation. Knockdowns of miR-155 reduced the effect that inflammatory cytokines had on glycolysis in breast cancer cells. MiR-155 was also found to promote pro-tumorigenic inflammatory STAT3 signaling by targeting (Suppressor of Cytokine Signaling 1) SOCS1, a repressor of Janus Kinase (JAK)/STAT signaling.

**Figure 2:**
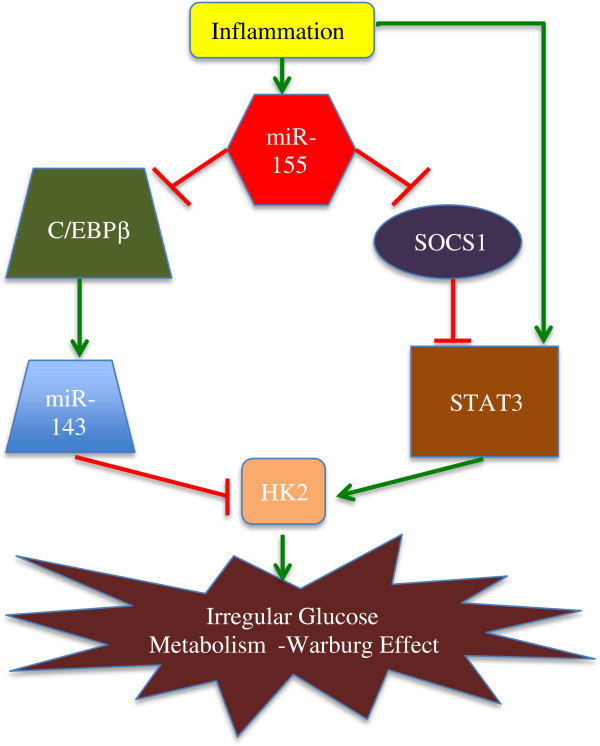
**The interplay of MiR-155 in inflammation and the Warburg effect of cancer [**[16]**].**

In summary, inflammation promoted glucose metabolism in cancer cells through miR-155-SOCS1-STAT3-HK2 and miR-155-C/EBPb-miR-143-HK2 cascades [16]. As miR-155 is upregulated after various inflammation stimuli, it forms an integral link between inflammation and cancer, as shown in Figure 2 below.

In their 2010 paper, Willimott and Wagner studied how miR-155 and miR-125b contributed to BCL2 repression and malignant cell proliferation [19]. They found that miR-155 and miR-125b repress BCL2 translation and mediate the proliferative response to CD40 ligand (CD154). CD40 signaling, in turn, promotes proliferation and rescues B-cells from apoptosis by inducing BCL2L1 and BCL2A1, while repressing BCL2. As CD154 drives mature B-cell malignancy proliferation, counteracting the effects of miRNAs induced by CD154 may have therapeutic benefits.

The addition of anti-miR-155 and anti-miR-125b prevented CD154-mediated repression of BCL2 and reduced CD154-mediated proliferation in the MEC1 B-cell line. CD-154 was found to increase miR-155 association with polysomes, while BCL2 was determined to be involved in chromosomal translocations in follicular lymphoma. MiR-155 was the most abundant miRNA under basal conditions [19]. This paper was important as it investigated the effect that miR-155 has on the important oncogene BCL2.

The preceding papers show that miR-155 interacts with miR-143 and miR-125b to promote malignant breast cell proliferation through the upregulation of inflammatory cytokine HK2 and tumor necrotic factor CD40. So far, we have seen how miR-155 causes cancer in most cases, prevents it in others, and associates with other microRNAs. The following section focuses on other important mechanisms of miR-155 action.

### Mechanisms of miR-155 action

In their 2009 *Nature Leukemia* paper, Forrest et al. studied how the induction of miR-155 promotes monocytic differentiation [18]. Their goal was to determine miRNAs that are regulated by phorbol myristate acetate (PMA), which is used to overcome a block in terminal differentiation of the myeloid lineage and progenitor state proliferation associated with AML in THP-1 cells. Along with miR-222, miR-424, and miR-503, miR-155 was the top PMA-induced miRNA, and it caused cell cycle arrest and partial differentiation when overexpressed.

This research showed that miR-155 plays a role in apoptosis by targeting anti-apoptotic factors such as RPS6KA3, SGK3, RHEB, and KRAS. MiR-155 was also determined to partially promote monocytic differentiation with selective depletion of myeloid and erythroid hematopoietic stem cell populations occurring in preference for B-cell proliferation. After 96-hour PMA-induced differentiation, THP-1 acute monolytic leukemia cells showed a 3-fold change in miR-155 expression through microRNA array analysis, and a 1.3-fold change in small RNA sequencing [18]. The data came from the FANTOM4 (Functional ANnoTation Of Mammals) project, which uses deep sequencing, bioinformatics predictions, microarrays, and siRNA perturbations to map a network of mammalian transcription factors and targets.

In the 2012 *PNAS* paper by Sandhu et al., the researchers sought insight into miR-155-induced leukemogenesis in Eμ-miR-155 transgenic mice. They did this through genome-wide transcriptome analysis of naïve B cells and target studies [8].

It was found that miR-155/BIC expression was negatively correlated with histone deacetylase 4 (HDAC4) and the transcriptional repressor and proto-oncogene, BCL6, in DLBCL patients. BCL-6 was downregulated in Eμ-miR-155 mice, while loss of miR-155 resulted in impaired immunity. This was due to defective T-cell-mediated immune response. BCL-6 was found to target the inhibitor of DNA-binding ID2, IL-6, cMyc, cyclin D1 and MiP1alpha/cd3, which all promote cell survival and proliferation. MiR-155 was determined to regulate BCL-6 through Mxd1/Mad1 upregulation. It also upregulated survival and proliferation genes (as noticed in miR-155-induced leukemias) and disrupted the BCL6 transcriptional machinery. Furthermore, miR-155 directly targeted HDAC4, a corepressor partner of BCL6. Ectopic expression of HDAC4 in human-activated DLBCL cells resulted in reduced miR-155-induced proliferation, clonogenic potential, and increased apoptosis. Tables 2 and 3 below show some of the pathways regulated by miR-155.

**Table 2:** Pathways up-regulated in Eμ-miR-155 mice naïve B cells

**Pathways**	**Molecules**
**AHR Signaling**	MGST1, CCNE2, NFIX, TFDP1, GSTM5, NQO2, POLA1, CDK6, CCND1, GSTO1, CHEK1, MYC, TGM2, CCNA2, CCND3, ALDH1A1, MGST2, E2F1, DHFR, ESR1, AHR [8]
**Glutathione Metabolism**	GSR, MGST1, MGST2, GSTM5, G6PD, IDH2, GCLM, GLRX, ANPEP, GSTO1, RNPEP, IDH1 [8]
**Mitotic Roles of Polio-like Kinase**	KIF23, PLK4, ESPL1, CDC20, PRC1, CCNB2, PLK1, CDK1, KIF11 [8]
**Communication between innate and adaptive immune cells**	TNFSF13, IL15, TLR7, FCER1G, Tlr13, IGHG1, CCL5, TLR3, CD8A, CCL9 [8]
**Role of pattern recognition receptors in recognition of bacteria and viruses**	IFIH1, CLEC7A, OAS1, C3, PIK3R6, TLR7, CCL5, EIF2AK2, TLR3, RNASEL [8]

**Table 3:** Pathways down-regulated in Eμ-miR-155 mice naïve B cells

**Pathways**	**Molecules**
**SAPK/JNK signaling**	GADD45A, DUSP4, MAP4K4, MAPK12, ATF2 [8]
**Activation of IRF by cytosolic pattern recognition receptors**	IKBKB, STAT2, MAPK12, ATF2 [8]
**Toll-like receptor signaling**	IKBKB, MAP4K4, MAPK12 [8]
**ATM signaling**	GADD45A, MAPK12, ATF2 [8]
**ERK/MAPK signaling**	ETS2, DUSP4, RPS6KA5, RAPGEF4, ATF2 [8]
**B-cell receptor signaling**	IKBKB, MAPK12, BCL6, ATF2 [8]

The previous studies show that miR-155 plays an integral role in numerous pathways. Specifically, miR-155 was shown to enhance AHR signaling, glutathione metabolism, mitosis, communication between innate and adaptive immune cells, and pattern recognition receptors. Meanwhile, miR-155 reduces IRF activation, and signaling for SAPK/JNK, toll-like receptors, ATM, ERK/MAPK, and B-cell receptors. Over the past few years, we have learned much about miR-155, its many mechanisms of action, and its interactions with other miRNAs. Combining this information with what we know about its role in cancer, we have also come up with promising new treatment options for patients.

### Anti-miR-155 treatment options

Traditional cancer therapy involves chemotherapy and radiation, which can lead to iatrogenic issues such as hair loss, decreased appetite and further mutations. However, some naturally occurring compounds such as curcumin, genistein, tea polyphenols, resveratrol, and sulforaphane seem to have anti-cancer properties [25-27]. In their groundbreaking Carcinogenesis paper, Tili et al. investigated the effect of resveratrol (trans-3,4′,5-trihydroxystilbene), a dietary polyphenolic, non-flavonoid antioxidant derived from grapes and berries, in inhibiting cancer [9]. They found that resveratrol impaired the lipopolysaccharide-induced upregulation of miR-155 in a way that depends on miR-663. Resveratrol was found to upregulate miR-663, which was found to decrease endogenous AP-1 activity and target jun-B and jun-D transcripts. Jun-B regulates gene activity following the primary growth factor response, and Jun-D protects cells from p53-dependent senescence and apoptosis.

Given miR-155′s role in oncogenesis, it would certainly be a suitable target for anti-miRs. However, free-floating anti-miRs are unstable in the plasma, must be efficiently delivered, and must overcome such obstacles as nucleases, non-specific tissue uptake, and renal clearance [28].

Recently, nanoparticle-based therapy has been shown to be effective against miR-155-dependent lymphoma in mice [15]. In research conducted at Yale, miR-155 overexpression in lymphoid tissues was shown to result in disseminated lymphoma with a clonal, transplantable pre-B-cell population of neoplastic lymphocytes*.* By contrast, miR-155 withdrawal by doxycycline resulted in regression of lymphadenopathy via apoptosis of malignant lymphocytes. When antisense peptide nucleic acids were delivered via unique polymer nanoparticles, miR-155 was inhibited and the growth of pre-B-cell tumors was slowed *in vivo*.

This research was important for two main reasons. Firstly, it confirmed that some lymphomas are miR-155 dependent. Secondly, it presented the most effective way to reverse the effects of miR-155 tumorigenicity *in vivo*, by nanoparticle delivery. This research was groundbreaking, as it paved the way for additional research with miR-155 and nanoparticles.

One such study took place at Ohio State University, where Zhang and fellow researchers used lactosylated gramicidin-containing lipid nanoparticles (Lac-GLN) to effectively deliver anti-miR-155 to hepatocellular carcinoma (HCC) cells [28]. The Lac-GLN formulation contained N-lactobionyl-dioleoyl phosphatidylethanolamine (Lac-DOPE) and an antibiotic peptide gramicidin A. Lac-DOPE is a ligand for the asialoglycoprotein receptor (ASGR).

While miR-155 expression was not affected, miR-155 target gene expression levels were upregulated in a dose-dependent fashion. It is believed that the delivery of anti-miR-155 blocked miR-155 function without facilitating its degradation. Low affinity binding of antisense oligonucleotide and its miRNA were determined to promote miRNA degradation, while high affinity binding repressed miRNA function [28].

This paper was among the first to report on a targeted lipid-based peptide system for anti-miRNA hepatic delivery [28]. The efficacy of this method was evidenced by the up-regulation of target genes that are repressed by miR-155, and is an area of follow-up study. Nevertheless, such research serves as a watershed in translating our knowledge about miR-155 into effective cancer therapy. Nanoparticle vehicles for anti-miR-155, particularly those that are lipid-based, hold promise for use in orthodox medicine, while resveratrol, and other naturally occurring agents [25-27], may be used to complement this in home-based or alternative treatment.

## Conclusions

This review outlines the role of miR-155 in oncogenesis. It targets numerous molecules in key signaling pathways such as glutathione metabolism, SAPK/JNK, TLR, ERK/MAPK, and B-cell receptor signaling. Because of its role in signaling pathways, miR-155 is a key contributor to cancers of the breast, lung, stomach, and particularly, the lymphatic system.

Some novel concepts that arose from the analysis of these papers were that miR-155 is not only a promoter of many cancers, but may act to prevent cancer in transgenic mice by promoting proper immune function. This finding conflicted what is generally known about miR-155 and may be attributable to the fact that the study was done in transgenic mice. Nevertheless this points to a need for more rigorous and applicable studies in humans, and to the vast effects of miR-155 on the immune system [29].

MiR-155 was also implicated in promoting apoptosis by targeting anti-apoptotic factors, and was seen to counteract miRNAs such as miR-663 and miR-143, and works along with miRNAs such as miR-125b and miR-146 [23]. Additionally, miR-155 targets important oncogenes such as BCL2, which regulates apoptosis, and BCL6, which represses transcription. It also counters kinases such as HK2, which phosphorylates glucose, thereby committing it to the glycolytic pathway. A recurring theme in current research is that miR-155 represses SHIP1, which serves an integral role in myeloid cell proliferation and survival. Cancer cells are notorious for their ability to evade apoptosis, and increase transcription and glucose metabolism [30,31]. With its combined role in preventing apoptosis, encouraging transcription, and blocking the phosphorylation of glucose, miR-155 is an obvious promoter of cancers, from lymphomas to mesenchymal malignancies such as liposarcoma.

This review outlined the emergence of novel treatment options, ranging from easily accessible naturopathic remedies such as resveratrol, to anti-TNF-α, and lipid nanoparticles. These new therapies hold promise for safer, more effective delivery cancer treatment in miR-155-dependent malignancies. Given the success of lipid nanoparticle delivery of anti-miR-155 in lymphoma and hepatocellular carcinoma cells (HCC), such delivery should be extended to other common sites of miR-155-dependent cancer, such as the breast and lungs. In translating treatment into clinics, the liver may be an easier target than most; it is a centralized organ through which all blood is filtered. The lymphatic system, which is the most common occurrence of cancers, may pose much more difficulty for targeting vectors, as it is decentralized.

Future research in this arena should be focused on improving the delivery of anti-oncomiRs to various tumor sites and on determining the effect of miR-155 in human tumors. Up to 25% of all cancers are due to chronic inflammation [32,33], while tumor metastasis is responsible for 90% of all cancer deaths [8]. As miR-155 has been linked to the development of lung and stomach tumors, agents that effectively block miR-155 will have incredible utility in cancer therapy.

## Abbreviations

AHR: Aryl hydrocarbon receptor; AID: Activation-induced deaminase; ALDH1A1: Aldehyde dehydrogenase 1 family, member A1; AML: Acute myeloid leukemia; ANPEP: Alanine aminopeptidase; AP-1: Activator protein-1; ASGR: Asialoglycoprotein receptor; ATF2: Activating transcription factor 2; BIC: B-Cell Integration Cluster; C-MAF: Musculoaponeurotic fibrosarcoma oncogene homolog; C/EBP-Beta: CCAAT/enhancer-binding protein beta; C3: Classical Complement Pathway C3-convertase; CCL5: Chemokine (C-C motif) ligand 5; CCNA2: Cyclin A2; CD40: Cluster of differentiation 40; CDC20: Cell-division cycle protein 20; CDK1: Cyclin-dependent kinase 1; CDK6: Cyclin-dependent kinase 6; CHEK1: Checkpoint kinase 1; CK1-α: Casein kinase 1-α; CLEC7A: C-type lectin domain family 7, member A; DHFR: Dihydrofolate reductase; DLBCL: Diffuse large B-cell lymphoma; DUSP4: Dual specificity protein phosphatase 4; E2F1: Higher Eukaryote Transcription Factor 1; EIF2AK2: Eukaryotic translation initiation factor 2-alpha kinase 2; ERK: Extracellular-signal-regulated kinases (ERKs) or classical MAP kinases; ESPL1: Extra spindle pole bodies homolog 1; ESR1: Estrogen Receptor 1; ETS-1: Erythroblastosis virus E26 oncogene homolog 1; ETS2: v-ets erythroblastosis virus E26 oncogene homolog 2 (avian); FANTOM: Functional ANnoTation Of Mammals; FCER1G: Fc fragment of IgE, high affinity 1 receptor for gamma polypeptide; FOXO3: Forkhead box O3; G6PD: Glucose-6-phosphate dehydrogenase; GADD45A: Growth arrest and DNA-damage-inducible, alpha; GCLM: Glutamate-cysteine ligase, modifier; GLRX: Glutaredoxin (thioltransferase); GSR: Glutathione reductase; GSTM5: Glutathione S-transferase mu 5; GSTO1: Glutathione S-transferase omega 1; HCC: Hepatocellular carcinoma cells; HDAC4: Histone deacetylase 4; HK2: Hexokinase 2; ID2: Inhibitor of DNA binding 2; IDH2: Isocitrate dehydrogenase 2 (NADP+), mitochondrial; IFIH1: Interferon induced with helicase C domain 1; IGHG1: Immunoglobulin heavy constant gamma 1 (G1m marker); IKBKB: Inhibitor of kappa light polypeptide gene enhancer in B-cells, kinase beta; IKK-ϵ: Inhibitor of nuclear factor kappa-B kinase subunit epsilon; IL-#: Interleukin-#; JAK/STAT: Janus Kinase/signal transducer and activator of transcription pathway BCL2, B-cell CLL/lymphoma 2; Jun-B: Jun B proto-oncogene; KIF11: Kinesin family member 11; KIF23: Kinesin family member 23; KRAS: Kirsten rat sarcoma viral oncogene homolog; Lac-DOPE: N-lactobionyl-dioleoyl phosphatidylethanolamine; Lac-GLN: Lactosylated gramicidin-containing lipid nanoparticles; MAP4K4: Mitogen-activated protein kinase kinase kinase kinase 4; MAPK12: Mitogen-activated protein kinase 12; MGST1: Microsomal glutathione S-transferase 1; MGST2: Microsomal glutathione S-transferase 2; MiP1-alpha: Macrophage inflammatory protein alpha; miR-155: microRNA-155; miRNA: microRNA; MLH1: MutL homolog 1; MMTV-PyMT: Mouse mammary tumor virus-polyoma middle T (antigen); MSH2/6: MutS protein homolog 2/6; MYC: V-myc myelocytomatosis viral oncogene homolog (avian); NFIX: Nuclear factor I/X; NQO2: NAD(P)H dehydrogenase, quinone 2; OAS1: 2′-5′-oligoadenylate synthetase 1; oncomiR: Oncogenic microRNA; PIK3R6: Phosphoinositide-3-kinase, regulatory subunit 1; PLK1: Polio-like kinase 1; PLK4: Polo-like kinase 4; PMA: Phorbol myristate acetate; POLA1: Polymerase (DNA directed) alpha 1; PRC1: Protein regulator of cytokinesis 1; RAPGEF4: Rap guanine nucleotide exchange factor 4; RHEB: Ras homolog enriched in brain; RHOA: Ras homolog gene family, member A; RNASEL: Ribonuclease L; RNPEP: Arginyl aminopeptidase (aminopeptidase B); RPS6KA3: Ribosomal protein S6 kinase, 90 kDa, polypeptide 3; SAPK/JNK: Mitogen-activated protein kinase 9, JUN N-terminal kinase; SGK3: Serum/glucocorticoid regulated kinase family, member 3; SHIP1: SH2 domain containing inositol-5-phosphatase; siRNA: Small interfering RNA; SOCS1: Suppressor of cytokine signaling 1; STAT2: Signal transducer and activator of transcription 2; STAT3: Signal Transducer & Activator of Transcription 3; TAM: Tumor-associated macrophage; TFDP1: Transcription factor dimerization partner 1; TGM2: Transglutaminase 2; THP-1: Tamm-Horsfall protein 1; TLR7: Toll-like receptor 7; TNF: Tumor necrotic factor; TNFSF13: Tumor necrosis factor (ligand) superfamily, member 13; TP53INP1: Tumor protein 53-inducible nuclear protein 1.

## Competing interests

The authors have no potential competing interests.

## Authors’ contributions

GH conducted the research and wrote the manuscript. FS edited the piece. Both authors read and approved the final manuscript.

## Authors’ information

GH is a PhD student in Computational Biology and Bioinformatics at Yale University. His research is focused on improving compound identification and quantification through the creation of mapping algorithms.

FS is a Professor of Molecular, Cellular & Developmental Biology at Yale University. His lab uses molecular, genetic, bioinformatics, and genomic approaches to understand microRNAs in development and disease.
